# Kaurenoic Acid Reduces Ongoing Chronic Constriction Injury-Induced Neuropathic Pain: Nitric Oxide Silencing of Dorsal Root Ganglia Neurons

**DOI:** 10.3390/ph16030343

**Published:** 2023-02-23

**Authors:** Tiago H. Zaninelli, Sandra S. Mizokami, Mariana M. Bertozzi, Telma Saraiva-Santos, Felipe A. Pinho-Ribeiro, Gabriele Inácio de Oliveira, Renata Streck, Eduardo J. A. Araújo, Nilton S. Arakawa, Sergio M. Borghi, Rubia Casagrande, Waldiceu A. Verri

**Affiliations:** 1 Laboratory of Pain, Inflammation, Neuropathy, and Cancer, Department of Pathology, Center of Biological Sciences, Londrina State University, Londrina 86057-970, Paraná, Brazil; 2 Department of Pharmaceutical Sciences, Center of Health Sciences, Londrina State University, Londrina 86039-440, Paraná, Brazil; 3 Department of Histology, Londrina State University, Londrina 86057-970, Paraná, Brazil

**Keywords:** neuropathic pain, chronic constriction injury, CCI, nitric oxide, analgesia, *Sphagneticola trilobata*, kaurenoic acid, potassium channels

## Abstract

Kaurenoic acid (KA) is a diterpene extracted from *Sphagneticola trilobata* (L.) Pruski. KA presents analgesic properties. However, the analgesic activity and mechanisms of action of KA in neuropathic pain have not been investigated so far; thus, we addressed these points in the present study. A mouse model of neuropathic pain was induced by chronic constriction injury (CCI) of the sciatic nerve. Acute (at the 7th-day post-CCI surgery) and prolonged (from 7–14th days post-CCI surgery) KA post-treatment inhibited CCI-induced mechanical hyperalgesia at all evaluated time points, as per the electronic version of von Frey filaments. The underlying mechanism of KA was dependent on activating the NO/cGMP/PKG/ATP-sensitive potassium channel signaling pathway since L-NAME, ODQ, KT5823, and glibenclamide abolished KA analgesia. KA reduced the activation of primary afferent sensory neurons, as observed by a reduction in CCI-triggered colocalization of pNF-κB and NeuN in DRG neurons. KA treatment also increased the expression of neuronal nitric oxide synthase (nNOS) at the protein level as well as the intracellular levels of NO in DRG neurons. Therefore, our results provide evidence that KA inhibits CCI neuropathic pain by activating a neuronal analgesic mechanism that depends on nNOS production of NO to silence the nociceptive signaling that generates analgesia.

## 1. Introduction

Neuropathic pain is a complex syndrome that results from a nerve fiber injury or dysfunction such as those that occur in diabetes, autoimmune diseases, infections, trauma, and trigeminal neuralgia [1,2,3,4]. Damage to conductive fibers causes disturbances of sensory and motor functions, which are characterized by pain and neuroinflammation [5,6]. Hyperalgesia (an increase in response to painful stimuli), paresthesia (tingling sensation), allodynia (pain resulting from a normally non-painful stimulus), and spontaneous pain are symptoms related to neuropathic pain [2,3,5,7,8]. Furthermore, studies show that in addition to these symptoms, the patients eventually develop depression, sleep disorders, and physical function impairments [9].

Neuropathic pain mechanisms are complex. There is the involvement of macrophages, glial cells (Schwann cells, astrocytes, microglia, and oligodendrocytes), T lymphocytes, and neutrophils, as well as oxidative stress, cytokines, chemokines, and growth factors. All these cells and mediators contribute to modulating axonal damage, elicit activation, and culminate in the neuropathic pain phenotype [5,7,10]. In the case of a peripheral nerve injury, these mechanisms induce plastic changes in the primary afferent sensory neurons, causing their sensitization, which is observed as pain. The activity of the primary afferent sensory neurons can be assessed in the dorsal root ganglia (DRG) since in this anatomical localization we can find the cellular bodies of those nociceptive neurons whose axons project to the peripheral tissues and spinal cord [1,7,10,11].

Several therapeutic drugs are available for controlling neuropathic pain, in general aiming to decrease neuronal activity, but without adequate effectiveness [12,13]. In fact, pharmacological management of neuropathic pain is considered to have a history of negative results in clinical trials [13,14]. Patients with neuropathic pain do not respond well to therapy with non-steroidal anti-inflammatory drugs, and resistance and tolerance to opiates are often observed. Contemporary tricyclic antidepressants and anticonvulsants are extensively used to treat neuropathic pain; however, all of them have limited effectiveness [13,14]. Furthermore, available therapies are often associated with side effects such as nausea, sedation, drowsiness, and dizziness [1].

Diterpenes are natural compounds with protective biological effects in various diseases [15,16]. One of these compounds, kaurenoic acid (KA), possesses a wide variety of activities. For instance, KA presents antitumor activity by activating apoptotic pathways [17,18,19] and reduces asthma inflammation [20]. Treatment with KA was demonstrated to be beneficial for the cardiovascular system by promoting antispasmodic and vasorelaxant actions, in part, by activating the NO-cGMP pathway [15,21]. Moreover, KA has analgesic properties since it inhibits inflammatory pain in pre-clinical models of acetic acid-induced and phenyl-p-benzoquinone-induced writhings, formalin- and complete Freund’s adjuvant (CFA)-induced paw flinching and licking, and carrageenan-, CFA- and LPS-induced mechanical hyperalgesia [22,23]. Those studies demonstrated that the analgesic mechanisms of KA depend, at least in part, on the reduction of pro-inflammatory cytokines and on the increase in NO-cGMP (cyclic guanosine monophosphate)-PKG (protein kinase G)-ATP-sensitive potassium channel signaling pathway [22,23,24,25]. This NO-dependent mechanism of KA in inflammatory pain seems to be of importance since activating the NO-cGMP-PKG-ATP-sensitive potassium channel is an analgesic mechanism of morphine, dipyrone, and other molecules with analgesic activity that activate this signaling pathway [26,27,28]. However, the effect and mechanisms of KA in neuropathic pain have not been investigated to our knowledge, nor has it been determined if KA activates a peripheral neuron NO-dependent mechanism to induce analgesia in neuropathic pain, which were the aims of the present study.

## 2. Results

### 2.1. KA Reduces CCI-Induced Mechanical Hyperalgesia in Mice

**Figure 1:**
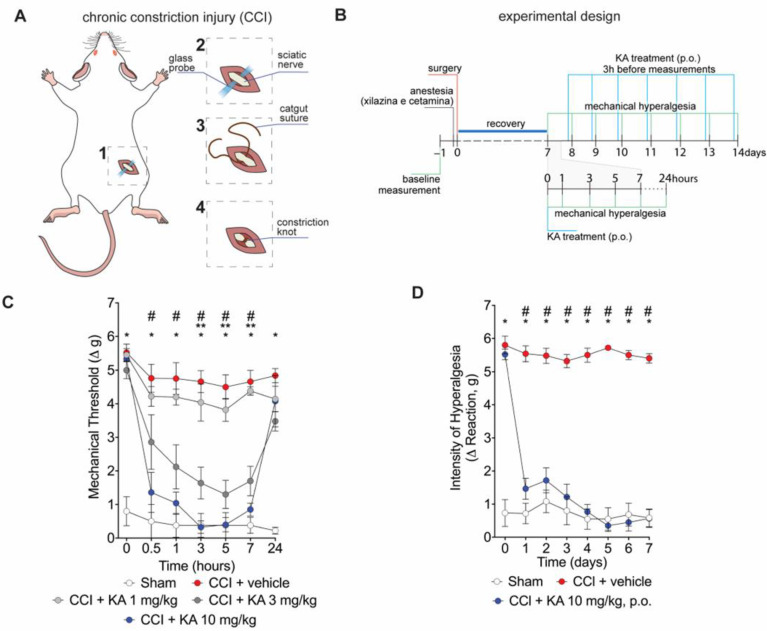
KA reduces CCI-induced mechanical hyperalgesia. (**A**) Scheme of the chronic constriction injury (CCI) model technique. (**B**) Scheme of experimental design for dose–response and KA chronic administration. (**C**) dose-dependent effects of KA treatment in mechanical hyperalgesia evaluated from 0–24 h, 7 days after surgery. (**D**) effects of KA chronic treatments (daily, 7 consecutive days, 7–14 days post-surgery). In the chronic treatment protocol measurements (**D**) were performed 3 h after KA administration. Results are presented as delta (Δ) of withdrawal threshold (in grams) calculated by subtracting the mean measurements at indicated time points from the basal mean measurements. Results are presented as mean ± SEM of measurements, *n* = 6 mice per experimental group (* *p* < 0.05 vs. sham, # *p* < 0.05 10 mg/kg vs. vehicle-treated group, ** *p* < 0.05 3 mg/kg vs. vehicle-treated group.

Mice underwent surgery for the induction of CCI (Figure 1A). After the recovery period (Figure 1B), mice were treated with KA or vehicle by per oral gavage (p.o.) in two treatment protocols. Firstly, mice were treated with KA in three different doses (1, 3, or 10 mg/kg) (Figure 1B). This dose range and route of administration were based on a prior study from our group [23]. Mechanical hyperalgesia was evaluated 0.5, 1, 3, 5, 7, and 24 h after treatment (Figure 1C). KA dose-dependently reduced CCI-induced mechanical hyperalgesia (Figure 1C). No significant effect was observed at the dose of 1 mg/kg. On the other hand, the dose of 3 mg/kg of KA reduced the mechanical hyperalgesia between 3 and 7 h, and the doses of 10 mg/kg reduced the mechanical hyperalgesia from 0.5 up to 7 h after treatment. Maximal analgesia was reached 3 h after KA administration at the dose of 10 mg/kg (Figure 1C). Thus, the dose of 10 mg/kg of KA and measurement of hyperalgesia 3 h after treatment were the parameters selected for the next experiments. For the chronic treatment protocol, mice were treated daily with KA (10 mg/kg, p.o.) for seven consecutive days, and the mechanical hyperalgesia was evaluated daily, always 3 h after the treatment (Figure 1D), since it was the peak of its analgesic effect (Figure 1C). KA inhibited CCI-induced mechanical hyperalgesia at all time points evaluated in the chronic treatment protocol (Figure 1D). To avoid misinterpretation, it is important to mention that comparing Figure 1C,D, the results of 24 h and 1 day time points, respectively, may seem contradictory. However, the explanation is that in Figure 1C, mice were treated once with KA, and hyperalgesia was measured up to 24 h. In Figure 1D, the one-day time point is not referring to the time after treatment with KA. In Figure 1D, mice received daily treatments with KA, and hyperalgesia was measured 3 h after each treatment. Thus, 24 h in Figure 1C and 1 day in Figure 1D do not represent the same time point after KA treatment.

### 2.2. The Analgesic Effect of KA in CCI-Induced Mechanical Hyperalgesia Depends on Activating the NO-cGMP-PKG-ATP-Sensitive Potassium Channel Signaling Pathway

**Figure 2:**
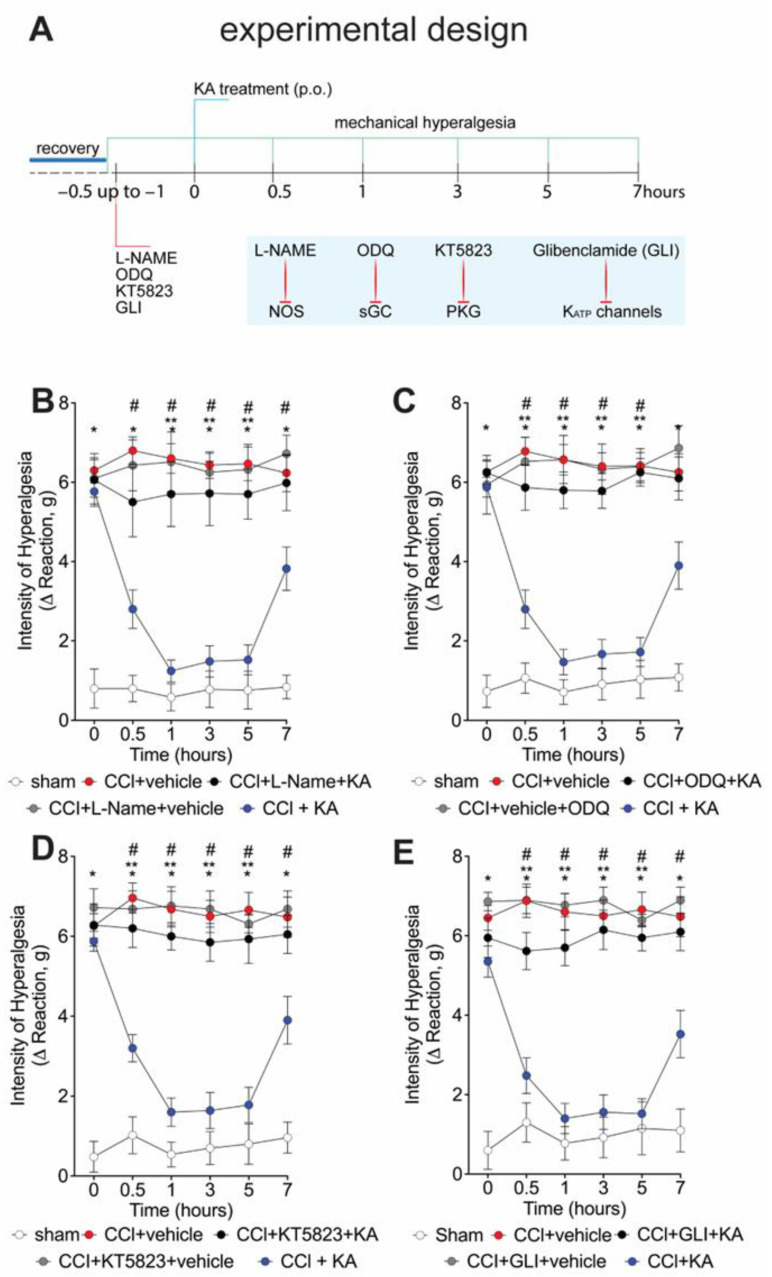
KA reduces CCI-induced mechanical hyperalgesia via the NO–cGMP–PKG–K_ATP_ channel signaling pathway activation. (**A**) Schematic representation of the experimental design for data acquisition. Mechanical hyperalgesia was evaluated seven days after CCI surgery (recovery). Mice were treated with vehicle, (**B**) **L**-NAME (NOS inhibitor; i.p., 90 mg/kg, 1 h), (**C**) ODQ (guanylate cyclase inhibitor; i.p., 0.3 mg/kg, 30 min), (**D**) KT5823 (PKG inhibitor; i.p., 0.5 μg/mice, 5 min), or (**E**) glibenclamide (K_ATP_ channel inhibitor; p.o., 0.3 mg/kg, 45 min), before KA treatment (p.o., 10 mg/kg). After additional 30 min, mechanical hyperalgesia was measured as per the electronic version of von Frey filaments from 0–7 h. Results are presented as delta (Δ) withdrawal threshold (in grams) calculated by subtracting the mean measurements at indicated time points from the basal mean measurements. Results are presented as mean ± SEM of measurements, *n* = 6 mice per group (* *p* < 0.05 vs. sham, # *p* < 0.05 vs. vehicle-treated group, ** *p* < 0.05 vs. CCI + KA-treated group.

We have previously shown that the analgesic effect of KA depends on activating the NO-cGMP-PKG-ATP-sensitive potassium channel signaling pathway in the carrageenan model of paw inflammation [23]. However, whether KA would induce analgesia in neuropathic pain by a similar mechanism was unknown. Thus, seven days after CCI, mice were pre-treated with **L**-NAME (NOS inhibitor), ODQ (soluble guanylate cyclase inhibitor), KT5823 (PKG inhibitor), or glibenclamide (ATP-sensitive potassium channel blocker) before the treatment with KA (10 mg/kg, p.o.) (Figure 2A). The mechanical hyperalgesia was evaluated 1–7 h after treatment with KA. Treatment with KA significantly reduced CCI-induced mechanical hyperalgesia at all evaluated time points (Figure 2B–E), which was reversed by the inhibitors **L**-NAME (Figure 2B), ODQ (Figure 2C), KT5823 (Figure 2D), and glibenclamide (Figure 2E). These results demonstrate that the anti-hyperalgesic effect of KA depends on triggering the NO-cGMP-PKG-ATP-sensitive potassium channel signaling analgesic pathway. It is important to mention that the selected treatment protocols with **L**-NAME, ODQ, KT5823, and glibenclamide have no effect per se [23], as we also confirmed herein (Figure 2B–E).

### 2.3. KA Reduces CCI-Induced Dorsal Root Ganglia (DRG) Activation

**Figure 3:**
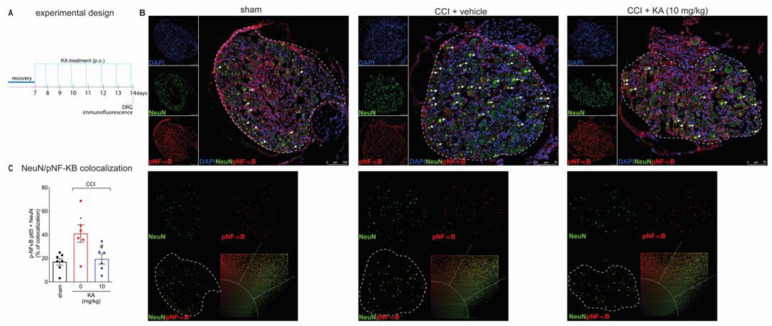
KA inhibits CCI-induced NF-κB activation (phosphorylated, p) in DRG neurons. (**A**) Schematic representation of the protocol used during the experimental design. (**B**) The DRGs (ipsilateral, vertebrae L_4_–L_6_) were dissected and processed for immunofluorescence assay for the different experimental groups. (**C**) Quantitative analysis for pNF-κB (p65)/NeuN colocalization area (dashed line) in percentage (%) complemented by scatterplot of each experimental group. All neurons in the dashed line area were considered. Results are presented as mean ± SEM of measurements, *n* = 6 mice per group. White arrows point to pNF-κB^+^NeuN^+^ stained neurons (* *p* < 0.05 vs. sham, # *p* < 0.05 KA 10 mg/kg vs. vehicle-treated group).

Peripheral nerve injury causes peripheral neuronal activation to trigger neuropathic pain [29,30]. The NO-cGMP-PKG-ATP-sensitive potassium channel signaling is a neuronal mechanism [31,32]. However, most studies investigating that pathway using pharmacological tools do not verify neuronal activation or even neuronal production of NO. The following series of experiments were designed to answer these questions to foster a mechanistic understanding of the KA mode of action in neuropathic pain. Our next step was to evaluate the effects of KA treatments on the dorsal root ganglia neurons’ activity at the level equivalent to the sciatic nerve. We stained phosphorylated (p)-NF-κB as a surrogate marker of cellular activation. DAPI was used as a nuclear marker to confirm the pattern of staining of NeuN, which is a neuronal marker present in the nucleus. The analysis of colocalization with pNF-κB was performed using only NeuN to restrict it to the neuronal population and exclude non-neuronal cells. With these markers, we analyzed their colocalization, which can serve to demonstrate active pNF-κB is in the nucleus of neurons to ascertain neuronal activation in CCI as well as the effect of KA treatment (Figure 3A,B). KA reduced the CCI-induced percentage of pNF-κB and NeuN colocalization areas (Figure 3C). This result suggests that one of the analgesic mechanisms of KA might be related to the decrease in pNF-κB translocation to the nucleus, thus reducing neuronal activation and pain.

### 2.4. KA Increases the Expression of Neuronal Nitric Oxide Synthase (nNOS) In Vivo and the Production of NO in DRG Neuron Culture (In Vitro)

**Figure 4:**
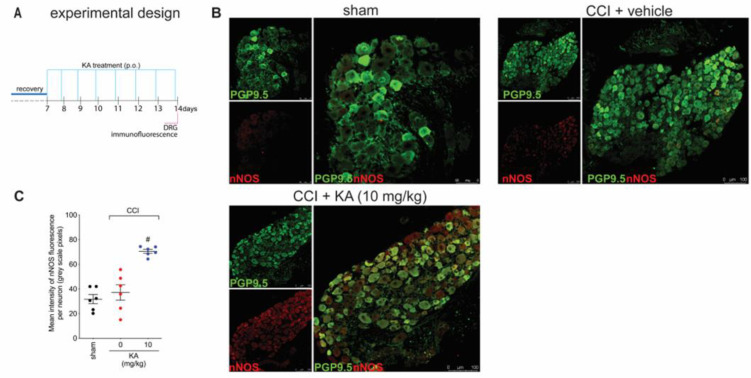
KA increases the expression of neuronal nitric oxide synthase (nNOS) in DRG neurons. (**A**) Schematic representation of the protocol used during the experimental design. (**B**) The DRGs (ipsilateral, vertebrae L_4_–L_6_) were dissected and processed for immunofluorescence assay for the different experimental groups. (**C**) Mean quantitative analyses of nNOS fluorescence intensity per neuron in DRG samples of 6 mice per group, ranging from 20–200 neurons per mouse. Results are presented as mean ± SEM of measurements, *n* = 6 mice per experimental group (# *p* < 0.05 vs. sham and vehicle-treated groups).

**Figure 5:**
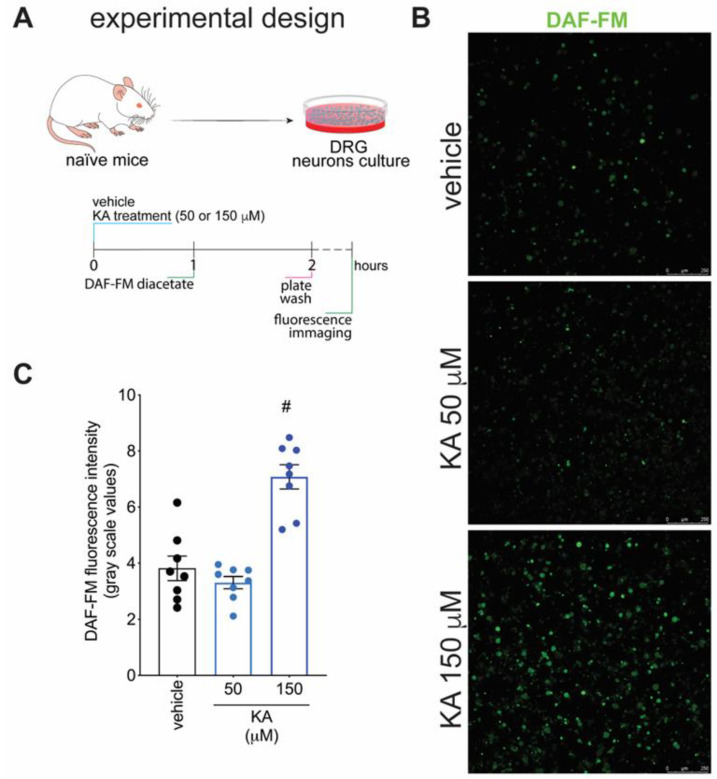
KA increases the intracellular levels of NO in DRG neurons in vitro. (**A**) Schematic representation of DRG neuron preparation and in vitro treatment protocols. (**B**) Cultured DRG neurons were treated according to the presented protocols and loaded with DAF-FM Diacetate (4-Amino-5-Methylamino-2′,7′-Difluorofluorescein Diacetate). (**C**) Quantitative analysis of DAF-FM fluorescence intensity (in grayscale values). Results are presented as mean ± SEM of measurements, *n* = 8 mice per experimental group (# *p* < 0.05 vs. vehicle-treated group).

Figure 1 demonstrates that KA has analgesic activity in neuropathic pain; Figure 2 demonstrates that the mechanism of KA depends on activating the NO-cGMP-PKG-ATP-sensitive potassium channel signaling; and Figure 3 shows that KA reduces the activation of the primary afferent neurons in CCI. Our next step was to verify whether KA could alter the staining for nNOS, which is a non-inflammatory source of NO, at a level where this endogenous gas functions as a signaling molecule, which is crucial to physiological and pathological events [33]. After 7 days of treatment with KA, DRG neurons were dissected and processed for neuronal nitric oxide synthases (nNOS) staining, and the mean intensity of staining per neuron in the ganglia of each mouse was quantitated (Figure 4A). PGP9.5 was used as a PAM neuronal marker. KA chronic treatment considerably increased the mean level of nNOS staining per neuron in the DRG ganglia of mice in the DRGs in comparison to sham or vehicle-treated CCI mice (Figure 4B,C). Then, our next step was to measure the effects of KA on the levels of NO in the DRG neurons. Using an in vitro approach (Figure 5A), treatment with KA increased intracellular levels of NO in a concentration-dependent manner (Figure 5B,C). Therefore, in the context of CCI neuropathic pain, KA treatment can increase the protein levels of nNOS (Figure 4). In addition to this mechanism, direct treatment with KA in cultured DRG neurons can induce NO production (Figure 5). Therefore, the present results are aligned with each other as well as bring novel evidence-based data to support that KA can induce analgesia by increasing neuronal NO production, together with the pharmacological and behavioral data of Figure 2. This experimental evidence also supports previous studies that used solely pharmacological tools to demonstrate that KA acts via neuronal NO production to induce analgesia [23].

## 3. Discussion

Neuropathic pain caused by CCI involves several mechanisms in peripheral and central systems, which pose major challenges in the development of fully effective therapeutic approaches. In peripheral nerve neuropathy, nerve injury increases neuronal activation and tissue inflammation which promote the release of neuropeptides and several pro-inflammatory mediators (e.g., cytokines, prostaglandin, and reactive oxygen species), respectively. Those events drive and maintain peripheral sensitization, neuroinflammation, and neuropathic pain [7]. Even with the evolving knowledge about the players in neuropathic pain, the nociceptive neurons are still a universally aimed target. Here, we demonstrate that KA treatment dose-dependently inhibits ongoing CCI neuropathic pain in mice. Furthermore, the analgesic effect of KA was maintained with daily treatment for seven days. In terms of mechanisms, the analgesic effect of KA lies in the (i) reduction of DRG neurons’ activation as demonstrated using pNF-κB colocalization with NeuN, (ii) increase in nNOS expression, and (iii) NO production by DRG neurons, which support the pharmacological evidence of dependency on activating a NO signaling analgesic neuronal mechanism.

The analgesic mechanism of KA was previously demonstrated using pharmacological tools to be dependent on the NO-cGMP-PKG-ATP-sensitive potassium channel signaling pathway in a model of carrageenan-induced inflammatory pain [23]. This was a starting point in our study since we wanted to advance in understanding the activity and mechanisms of KA. We identified that although pharmacological tools have been used and, overall, their activity is a reliable source to understand the mechanism of action of drugs, the literature was missing a deeper understanding of the importance of NO to the KA mechanism of action, specifically, nNOS-derived NO with a signaling role instead of iNOS-derived NO with a more robust production of NO and a role as a microbicidal macrophage molecule, as already explored before [34]. We observed that KA acute treatment could reduce CCI mechanical hyperalgesia as well as that a prolonged 7-day treatment could reduce pain. Similar to what has been demonstrated in inflammatory pain, KA analgesia in neuropathic pain was also dependent on the NO-cGMP-PKG-ATP-sensitive potassium channel signaling pathway, according to data obtained using pharmacological tools and behavior investigation.

As triggering the NO-cGMP-PKG-ATP-sensitive potassium channel signaling pathway is an analgesic neuronal mechanism, we started by investigating the activity of KA over DRG neuron activation. NF-κB has a crucial role in inflammation [35], and pain in inflammatory and neuropathic conditions [36,37]. Since the cellular bodies of nociceptor neurons—a subset of sensory neurons specialized in pain signaling—are in the DRG, we evaluate the effects of KA on NF-κB phosphorylation and translocation to the nucleus in this tissue. Our findings demonstrate that KA treatments reduced the translocation of phosphorylated NF-kB to the nucleus of DRG neurons, which suggests a reduction in nociceptor neuron activation [38]. We performed colocalization analyses using confocal microscopy and triple staining with pNF-κB, DAPI (a nuclear material marker) and NeuN (a neuronal nuclear marker [39]). These markers allowed to observe that CCI increases the neuronal nuclear localization of pNF-κB since NeuN localization is in the nucleus of neurons [39], and KA inhibits the colocalization of pNF-κB and NeuN. Previous studies have observed that KA can reduce the activation of NF-κB; however, this was demonstrated in leukocytes [22,23] and not in neurons as in the present study; thus, we add a novel cellular target for KA. In pre-clinical models of CFA-, carrageenan-, and LPS-induced inflammatory pain, KA treatment reduced events that are regulated or that activate NF-κB to induce pain such as cytokine production and oxidative stress [22,23], which are likely occurring mostly in immune cells like macrophages [34]. In addition, in a model of acetic acid-induced colitis, KA reduced neutrophil infiltration and lipid peroxidation [40]. These data further support the relevance of KA as a potential treatment for inflammation [40], inflammatory pain [22,23], and now neuropathic pain.

Although pharmacological tools have allowed to identify that KA analgesia in inflammatory [23] and neuropathic pain (present data) depend on activating the NO-cGMP-PKG-ATP-sensitive potassium channel signaling pathway, it has not been previously demonstrated that KA induces NO production by neurons. In this study, we have shown for the first time that KA treatment increases the expression of nNOS in DRG neurons during CCI neuropathy, which supports that KA analgesia would depend on NO production. Moreover, a single treatment with KA increased the intracellular levels of NO in nociceptor neurons from naïve mice DRG. The activity in cultured DRG neurons is important since it demonstrates that KA has direct neuronal effects, which have not been previously demonstrated. However, we must mention that our study has the limitation of not measuring NO in vivo in the CCI model, which is a methodologically challenging task. Nevertheless, we can suggest that, to some extent, the DRG neuronal culture may have some resemblance to a neuropathic condition despite having been collected from naïve mice. This suggestion is based on the fact that to isolate the DRGs, there is an axotomy process, which is a procedure that induces neuropathic pain if performed in vivo [41]. Our results support the hypothesis that, acutely, KA can induce neuronal NO production and the consequent effects (e.g., analgesia), and in a repetitive treatment approach, KA might also enhance NO production by up-regulating nNOS. NO and other forms of NO with different charges such as nitroxyl/HNO, or substances that increase the neuronal concentration of NO also cause analgesia by activating the cGMP-PKG-ATP-sensitive potassium channel signaling pathway [28]. In fact, NO can stimulate cGMP production, leading to the activation of PKG, which phosphorylates the ATP-sensitive potassium channel. Upon opening of potassium channels, there is hyperpolarization of nociceptive neurons, reducing the depolarization and action potential transmission, thus, promoting the silencing of nociceptor neurons, which is translated as analgesia [42].

KA presents low cytotoxicity, and it is not genotoxic in vitro, as demonstrated in the culture of CHO-K1 cells [43]. Although KA has hemolytic activity in vitro, it occurs at high concentrations, with an IC50 of 100 mM [44]. Other studies report that cell viability is still maintained in most published studies with KA concentrations <200 μM [34,43,45]. For example, in a culture of human normal lymphocytes, KA at the concentration range of 7.8 to 500 µM did not affect cell viability over 24 h incubation period. However, upon long exposure periods (≥72 h) in cancer cell lines (MCF-7, HCT-8, and CEM), KA at 78 μM has been described to induce cytotoxicity [46]. Thus, KA seems to have a cytotoxicity that is selective to tumor cells, which does not occur at the same concentration range for normal cells. Considering that concentrations lower than 200 μM of KA seem to be safe for normal cells [34,43,45,46] and also that KA analgesia starts within 30 min after its in vivo administration, we have chosen the concentration range of 50–150 μM and an incubation period of 1 h to test its effect on inducing NO production. From the in vivo perspective, in a toxicity study, treatment of Swiss mice with an extract of *Wedelia paludosa* (Acmela brasiliensis) (Asteraceae), which is rich in KA, neither acute nor chronic exposure induced changes in weight, hematological, kidney, or liver parameters [25,47]. Moreover, KA treatment did not induce liver or stomach toxicity [23]. The parameters analyzed were the plasma levels of aspartate transaminase (AST) and alanine transaminase (ALT), for liver toxicity, and myeloperoxidase activity in the stomach [23]. It is also interesting to mention that in an animal model of acetaminophen-induced hepatotoxicity, treatment with KA (30 mg/kg) increased survival by reducing hepatic necrosis, ALT and AST levels, inflammation, and oxidative stress [48]. Therefore, in addition to not presenting side effects that are common to non-steroidal anti-inflammatory drugs, KA can treat an organ lesion caused by a toxic dose of a widely used analgesic drug. KA at the same treatment regimen as in the present study (10 mg/kg, p.o.) did not alter the ability of Swiss mice in the rota-rod apparatus. Thus, supporting KA has analgesic activity and not muscle relaxant or sedative activities that would avoid the nociceptive response by the mice [23].

## 4. Materials and Methods

### 4.1. Animals

Male Swiss mice (20–25 g), from the Londrina State University, Londrina, Paraná, Brazil, were used in this study. Mice were housed in standard clear plastic cages with free access to food and water and a light/dark cycle of 12:12 h. All behavioral testing was performed between 9 a.m. and 5 p.m. in a temperature-controlled room (21 °C). Animal care and handling procedures were in accordance with the International Association for the Study of Pain (IASP) guidelines and approved by the Ethics Committee of Londrina State University (process number 12105.2012.67, approved on 31 May 2012).

### 4.2. Extraction and Isolation of Kaurenoic Acid

The roots of *Sphagneticola trilobata* were collected at Horto de Plantas Medicinais of Londrina State University (23°19′41″ S/51°12′14″ W), in September 2011. A voucher specimen was deposited at the Londrina State University Herbarium (FUEL Herbarium) under number 49306, collected by one of the authors (Nilton S. Arakawa)**.** The voucher specimen was identified by Prof. Dra. Ana Odete Vieira, Department of Vegetable and Animal Biology, Londrina State University. The air-dried roots of *Sphagneticola trilobata* were pulverized and then extracted exhaustively with dichloromethane (900 mL) at room temperature, to give 1.2 g of crude extract, which was suspended in 300 mL of methanol−H_2_O (9:1 *v*/*v*) and filtered. The soluble fraction was partitioned using *n*-hexane (300 mL, four times), which resulted in a 0.7 g *n*-hexane-soluble fraction after solvent evaporation under reduced pressure. The *n*-hexane-soluble fraction was chromatographed over silica gel 60 (0.063−0.200 mm) using vacuum-liquid chromatography with *n*-hexane and increasing amounts of ethyl acetate as eluents (250 mL of each fraction). The second fraction (0.41 g) was washed with cold methanol, to afford Kaurenoic acid (*ent*-kaur-16-en-19-oic acid; 800 mg, purity 96%, as determined by HPLC), exhibiting [*α*]^20^_D_–110, similar to a previous report [49]. EIMS *m/z* 325 [M + Na]^+^ and compared by ^1^H (CDCl3, 400 MHz) and ^13^C (CDCl3, 100 MHz) spectroscopy with an authentic standard and literature data [50].

### 4.3. Drugs and Stimuli

The following materials were obtained from the sources indicated: Dimethyl sulfoxide (DMSO), N^G^-nitro-**l**-arginine methyl ester (**l**-NAME), and glibenclamide were obtained from Sigma-Aldrich (St. Louis, MO, USA). 2,3,9,10,11,12-hexahydro-10R-methoxy-2,9-dimethyl-1-oxo-9S,12R-epoxy-1diindolo [1,2,3-fg:3′,2′, 1′-kl] pyrrol [3,4-i][1,6]benzodiazocine-10-carboxylic acid, methyl ester (KT 5823) from Cayman Chemical Company (Ann Arbor, MI, USA), 1H-(1,2,4)-oxadiazolol-(4,3-a)quinoxalin-1-one (ODQ) was obtained from Santa Cruz Biotechnology (Santa Cruz, CA, USA), ketamine and xylazine from Sespo Industria e Comércio Ltd.a (Paulinia, SP, Brazil), and DAF-FM diacetate from Invitrogen (Waltham, MA, USA).

### 4.4. Model of Chronic Constriction Injury (CCI)

Mice were anesthetized with ketamine and xylazine (1:1; 10 μL/10 g of body weight, intramuscular), followed by trichotomy and asepsis of the surgery area. The surgical procedure was performed as previously described [51] with some modifications [10]. Briefly, an incision was performed in the rear leg, and the distal portion of the sciatic nerve was tied with surgical thread (catgut 4-0). For the sham controls, mice underwent the same procedure without the constriction of the nerve.

### 4.5. Experimental Protocols

The pain threshold to the mechanical stimulus was measured before the surgery (basal response) and at the following intervals, starting from the 7th day after CCI (peak of mechanical hyperalgesia) [10], 0.5–24 h or daily 3 h after per oral (p.o.) treatment with KA (1, 3, or 10 mg/kg/day, diluted in 2% DMSO in saline) or vehicle (2% DMSO in saline). To investigate the role of the NO-cGMP-PKG-ATP-sensitive potassium channel signaling pathway in the analgesic effect of KA, the following drugs were administered before KA (10 mg/kg, p.o.) treatment: **L**-NAME [**L**-nitro-arginine methyl ester, NOS inhibitor, 90 mg/kg, intraperitoneal (i.p.), diluted in saline, 45 min), ODQ (soluble cGMP inhibitor; 0.3 mg/kg, i.p., diluted in 2% DMSO in saline, 30 min), KT5823 (PKG inhibitor; 0.5 µg/mice, i.p., diluted in 2% DMSO in saline, 5 min) and glibenclamide (ATP-sensitive potassium channel blocker; 0.3 mg/kg, p.o., diluted in 5% of Tween 80 in saline, 30 min). The effects of **L**-NAME, ODK, KT5823, and glibenclamide were also tested in vehicle-treated CCI-bearing mice. Upon results demonstrating that KA’s analgesic effect depends on a NO mechanism, animals were treated with KA or vehicle for 7 consecutive days. On the 7th day, dorsal root ganglia (DRG, L4-L6) were dissected and processed for immunofluorescence staining for neuronal activation (NeuN+pNF-kB+) and the expression of neuronal (PGP9.5) nitric oxide synthase (nNOS). The effects of KA on inducing neuronal production of nitric oxide were also evaluated in vitro using primary DRG culture imaging after incubation with the DAF-FM diacetate probe. The selected doses of drugs and time of sample collection were chosen based on pilot studies [28]. This also includes the doses and times of pre-treatment with **l**-NAME, ODQ, KT5823, and glibenclamide, which do not present an effect per se at these protocols, as we have already demonstrated [23].

### 4.6. Mechanical Hyperalgesia

The mechanical hyperalgesia test consisted of evoking a hind paw flexion reflex with a hand-held force transducer (electronic anesthesiometer; Insight, Ribeirao Preto) adapted with a 0.5 mm^2^ polypropylene tip, as previously described [10]. The endpoint was characterized by the removal of the paw followed by clear flinching movements. After the paw withdrawal, the intensity of the pressure was automatically recorded, and the value for the response was obtained by averaging three measurements. The animals were tested before and after the surgery and treatments. The results are expressed as delta (∆) withdrawal threshold (in g), calculated by subtracting from basal (before the surgery) and the mean of measurements obtained at 0.5–24 h (on the 7th-day post-CCI surgery), or 1–7 days (equivalent to 7–14 days post-CCI surgery) 3 h after the treatments with KA.

### 4.7. Immunofluorescence Staining

After surgical recovery, animals were treated with KA or vehicle for 7 consecutive days. On the 7th day, the DRGs corresponding to the right L4-L6 vertebrae were dissected and placed in 4% paraformaldehyde (*m*/*v*) in phosphate-buffered saline (PBS) at 4 °C for 24 h. After fixation, samples were dehydrated in a 30% sucrose solution in PBS (*m*/*v*) at 4 °C for 24 h. DRGs were frozen in Optimal Cutting Temperature (OCT). The samples were then sectioned in a cryostat at 15 mm of thickness and placed on a charged glass slide for immunofluorescence staining. Briefly, the sections were hydrated in PBS and blocked with a solution of 5% bovine serum albumin (*m*/*v*), 0.3% Triton 100-X (*v*/*v*) in PBS. Samples were incubated with mouse anti-mouse phospho-NF-κB (1:200, cat. # sc-136548, Santa Cruz Biotechnology [RRID:AB_10610391]) and rabbit anti-mouse NeuN (1:300, cat. # ABN78, Millipore Sigma [RRID: AB_10807945]), followed by goat anti-mouse Alexa Fluor 647 (1:500, cat. # A-21236, Thermo Fisher Scientific [RRID:AB_2535805]) and goat anti-rabbit Alexa Fluor 488 (1:500, cat. # A-11008, Thermo Fisher Scientific [RRID:AB_143165]), respectively. In another set of experiments, sections of DRGs were incubated with mouse anti-mouse PGP9.5 (1:750, cat. # ab8189, Abcam [RRID: RRID:AB_306343]) and goat anti-mouse nNOS (1:300, # GTX89962, GeneTex [RRID:AB_10725945]) followed by goat anti-mouse Alexa Fluor 594 secondary antibody (1:500, cat. # A21125, Thermo Fisher Scientific [RRID:AB_141593]) and donkey anti-goat Alexa Fluor 647 (1:500, cat. # A32849, Thermo Fisher Scientific [RRID:AB_2762840]), respectively. Image acquisition and analysis of fluorescence intensity were performed using a confocal microscope (TCS SP8, Leica Microsystems, Mannheim, Germany). LAS X software (Leica Microsystems, Mannheim, Germany) was used to determine the colocalization area of the neuronal nuclear markers NeuN (green) and p-NF-κB (red), which was normalized by the percentage of ganglia area (dashed line). DAPI was not used for this analysis. All fluorescence intensities were measured in grayscale values using the same software.

### 4.8. Intracellular Nitric Oxide Detection

The DAF-FM diacetate fluorescent probe (Invitrogen, Waltham, MA, USA) was used to determine the levels of nitric oxide in DRG neuron cultures. DRG were dissected from naïve Swiss mice, digested, and seeded on Nunc™ Glass Bottom Dishes at a density of 1 × 10^6^ per mL (1 × 10^5^/plate). Plates were incubated overnight in neurobasal medium, as previously described [38,52]. Neurons were treated either with vehicle (2% DMSO in culture media) or KA (50 and 150 μM) for 1 h at 37 °C and 5% CO_2_ atmosphere. The concentrations of KA were based on previous in vitro studies [34,43,45,53]. DAF-FM diacetate probe was added to the culture and further incubated for 1 h in neurobasal medium at 37 °C and 5% CO_2_ atmosphere. Plates were washed with warm Hank’s Balanced Salt Solution (HBSS), and the levels of NO were determined by confocal microscopy (TCS SP8, Leica Microsystems) with a 63× magnification objective. Results are presented as DAF-FM fluorescence intensity per imaged field.

### 4.9. Statistical Analyses

The sample size was determined using G*Power software (version 3.1.9.7, Düsseldorf, Germany) [54]. All data passed normality and homogeneity tests using the Shapiro–Wilk and Brown–Forsythe tests, respectively. Therefore, it was analyzed by one- or two-way analysis of variance (ANOVA) followed by Tukey’s post hoc. One-way ANOVA was used to analyze data within a single time point, while two-way ANOVA was used to compare groups and doses at all time points investigated. These data are presented as the mean ± standard error of the mean (SEM). Statistical differences were significant at *p* < 0.05.

## 5. Conclusions

In conclusion, KA reduces CCI neuropathic pain. The mechanisms of KA involve the silencing of nociceptive neurons, whose activity was diminished as observed by the reduction of colocalization data of pNF-kB and NeuN in DRG neurons. This silencing of nociceptive neurons involves the activation of the NO-cGMP-PKG-ATP-sensitive potassium channel signaling pathway, as observed using pharmacological tools. Corroborating with this pharmacological approach, KA enhanced nNOS staining in DRG neurons in CCI as well as induced NO production by cultured DRG neurons. Therefore, we demonstrate for the first time that KA is analgesic in neuropathic pain by mechanisms dependent on neuronal silencing via NO neuronal production that will ultimately activate the ATP-sensitive potassium channel, inducing neuronal hyperpolarization.

## Data Availability

Data is contained within the article.
